# Does direct farm marketing fulfill its promises? analyzing job satisfaction among direct-market farmers in Canada

**DOI:** 10.1007/s10460-021-10289-9

**Published:** 2022-01-31

**Authors:** Stevens Azima, Patrick Mundler

**Affiliations:** Department of Agri-Food Economics and Consumer Sciences, Université Laval, 2425 rue de l’Agriculture, Quebec, QC G1V 0A6 Canada

**Keywords:** Direct farm marketing, Intermediated food channels, Job satisfaction, Short food supply chains

## Abstract

Short food supply chains have become the focus of considerable research in the last two decades. However, studies so far remain highly localized, and claims about the economic and social advantages of such channels for farmers are not backed by large-scale empirical evidence. Using a web survey of 613 direct-market farmers across Canada, this article explores the potential economic and social benefits that farmers derive from participating in short food supply chains. We used multivariate analysis to test whether a farmer’s degree of involvement in direct food channels is positively correlated with levels of work enjoyment, social satisfaction, and economic satisfaction. The results indicate that, overall, direct-market farmers report high levels of occupational satisfaction, although work-related challenges persist, such as stress, excessive workloads, and competition. Farmer participation in short food chains was also a positive predictor of work enjoyment and economic satisfaction, but not of social satisfaction, as measured by the share of total farm sales attributable to direct selling. Net annual farm revenue, the share of direct food sales involving a middleman, age, and gender also correlated with one or more dimensions of occupational satisfaction.

## Introduction

Short food supply chains (SFSCs)^1^ are becoming increasingly popular as a way for consumers to purchase local food, and many countries have implemented policies to facilitate their development (Kneafsey et al. 2013; Rahe et al. 2019; Fardkhales and Lincoln 2021). Since SFSCs involve few or no middlemen, participating farmers are often more autonomous and have a greater ability to set prices independently (European Commission 2013). A growing number of studies over the last two decades have also examined the rise and organization of SFSCs (Venn et al. 2006; Tregear 2011; Kneafsey et al. 2013; Corsi et al. 2018; Dimitri and Gardner 2019; Paciarotti and Torregiani 2020) with much of this research focused on measuring their economic, social, and environmental impact (Schönhart et al. 2009; Martinez et al. 2010; Uematsu and Mishra 2011; Brunori et al. 2016; Vittersø et al. 2019).

Academic interest in SFSCs is occurring at a time when the industrial food system is coming under increasing criticism for its negative impact on the environment, producers, farmworkers, and communities (Hendrickson and Heffernan 2002; Jaffe and Gertler 2006; Weis 2007). Against this backdrop, SFSCs have emerged as “sites of resistance” to conventional agri-business (Campbell 2009) by offering consumers a “promise of difference” (Le Velly 2019). Presented as more virtuous, these channels are also perceived as facilitating a transition toward a more sustainable food system (Beus and Dunlap 1990; Kloppenburg et al. 2000; Constance et al. 2014). Yet, the notion that conventional and alternative food channels exist in separate spaces is contested by numerous scholars who point to the growing “conventionalization” of SFSCs and the emergence of hybrid initiatives that encompass aspects of both systems (Hinrichs 2000; Allen et al. 2003; DuPuis and Goodman 2005; Ilbery and Maye 2005; Sonnino and Marsden 2006). Promoters of direct marketing channels are also under growing pressure to scale up their impact (Friedmann 2007; Mount and Smither 2014; Zwart and Mathijs 2020). As such, the social, environmental, and economic benefits associated with SFSCs, while real, ought to be interpreted with caution.

Many studies on direct marketing analyze farm-level impacts and suggest, with varying degrees of evidence, that SFSCs generate economic and social benefits for participating farmers. Indeed, compared to conventional marketing, direct-farm sales can lead to better economic outcomes for producers, such as higher and more stable prices, enhanced revenues, greater market opportunities, and lower risk exposure (Govindasamy et al. 1999; Richard et al. 2014; Paul 2019). However, other researchers have questioned the extent of these benefits, arguing that positive findings often overlook the costs of participating in SFSCs (Hardesty and Leff 2010). Likewise, recent findings have produced mixed results regarding the impact of direct farm marketing on price setting and revenue levels (Mundler and Jean-Gagnon 2020).

From a social standpoint, farmers involved in SFSCs build positive connections with other community food stakeholders, such as local consumers and other farmers, leading to the creation of trust and social capital (Kneafsey et al. 2013). Studies have also highlighted the specific benefits of direct selling for women farmers (Tijani and Yano 2007; Zirham and Palomba 2016; Ball 2019). Despite these positive findings, SFSCs are not exempt from certain criticisms made of conventional supply chains, such as unequal power relations among farmers and between farmers and consumers (Hinrichs 2000; DuPuis and Goodman 2005). Indeed, competition among direct-market farmers can even stifle efforts at cooperation (Connolly and Klaiber 2019).

While direct farm marketing has garnered increasing attention among scholars, most of the research to date involves “highly localized case studies” (Venn et al. 2006, p. 253; Kneafsey et al. 2013), which do not offer a widescale assessment of the socio-economic impact of SFSCs on farmers. Further complicating matters is the fact that direct-market producers commonly use conventional distribution chains to varying degrees (Brown and Miller 2008). Nevertheless, despite a growing awareness of the “hybrid” nature of modern-day food systems (Ilbery and Maye 2005), researchers have generally failed to account for the diversity of marketing strategies pursued by farmers.

Likewise, it remains unclear how activities associated with direct marketing (such as processing, distribution, and sales) affect productivity, social relationships, and the nature of farm work (see Mundler and Jean-Gagnon 2020). Also missing from the literature on SFSCs is an analysis of the physical and psychological factors that shape the farming experience. After all, farming is a physically-demanding profession, with producers often exposed to numerous work-related psychological stressors (Deary et al. 1997; Fraser et al. 2005). Despite these difficulties, farmers often report that working in agriculture is an enjoyable experience that gives them satisfaction and a sense of pride (Coughenour and Swanson 1988).

Recent scholarship on SFSCs has begun to explore farm work from the viewpoint of occupational satisfaction (Mundler and Laughrea 2016; Dumont and Baret 2017; Dupré et al. 2017). The topic is an important one given that direct marketing has long been presented as a means for family farms to stay afloat by circumventing an industrial food system that contributes to declining agricultural revenues and producer autonomy (Renting et al. 2003). While farming is in many ways a unique profession, much of the literature on job satisfaction is potentially applicable (Aziri 2011). Moreover, a sociological framework developed by Paugam (2000) has already been successfully used to qualitatively explore levels of reported satisfaction among direct-market farmers (Dufour et al. 2011). Following Paugam (2000), we examine the impact of SFSCs on farmers using three key dimensions of occupational satisfaction: (1) work enjoyment (the farmer as *homo faber*), (2) social satisfaction (the farmer as *homo sociologicus*), and (3) economic satisfaction (the farmer as *homo economicus*).

With the help of different measurement tools, the proposed framework can be used to determine the occupational benefits of farmer involvement in SFSCs. So far, the geographical scope of studies utilizing this approach has been limited, and no attempt has been made to model the relation between the degree of SFSC participation and farmer satisfaction. Our research addresses this gap in the literature by using data from a research project conducted in 2019 involving a sample of 613 direct-market farmers from across Canada. We use a multifaceted approach to investigate whether increased participation in SFSCs is correlated with greater levels of satisfaction among farmers. To our knowledge, this is the first article that examines the country-wide occupational impact of direct farm marketing. In so doing, we seek to inform the debate around the social and economic advantages of SFSCs.

The rest of the article is organized as follows. We first describe the analytical framework used to measure job satisfaction, incorporating existing research on the benefits of direct marketing. We then present our methodology, followed by the results. Finally, we conclude by discussing how our findings contribute to existing research on SFSCs.

## Short food chains and farmer satisfaction

### Occupational satisfaction in agriculture: an overview

Work satisfaction is a topic of interest for policymakers, the media, as well as researchers in various disciplines (management, economics, psychology, sociology) who have proposed different ways of defining and measuring the concept. While a variety of job satisfaction models exist, one of the most widely tested has been Hackman and Oldham’s (1974) job characteristics model. It posits that satisfaction is positively related to five main occupational features:

- skill variety (the variety of activities required)
- task identity (the extent to which the work is meaningful)
- task significance (how the work affects the lives or work of others)
- workplace autonomy
- performance feedback

According to numerous studies and meta-analyses, these job features have a significant impact on occupational satisfaction. Furthermore, it has been shown that satisfaction levels can improve through organizational interventions aimed at improving the work environment (Loher et al. 1985; Blanz 2017). Overall, these results suggest that certain occupational characteristics linked to SFSC participation might correlate with increased levels of farmer satisfaction.

Job satisfaction is commonly defined as the way “people feel about their jobs and different aspects of their jobs, the extent to which people like (satisfaction) or dislike (dissatisfaction) their jobs” (Spector 1997, p. 2). The concept is multifaceted and usually measured using a single Likert item to determine overall satisfaction or through a series of Likert items that explore different work aspects (D’Addio et al. 2007). Previous research has found that satisfaction scores for single- and multi-item questions tend to converge, although discrepancies can remain (Wanous et al. 1997). As Cabrita and Perista (2006, p. 8) put it: “the reported overall job satisfaction may capture some additional aspects of the jobs held or reflect differences in the weight each employee attaches to individual job facets. In other words, the overall rating for job satisfaction is not likely to be a simple average of the workers’ satisfaction levels for the different aspects of a job but will be a more complex assessment.”

While occupational satisfaction has been studied extensively across various disciplines, the models developed so far usually focus on workers within hierarchical organizations and, as such, are poorly adapted to the world of farming and even less so to direct marketing. Farming is considered a high-risk occupation, characterized by high rates of depression and suicide (Deary et al. 1997; Pickett et al. 1999; Fraser et al. 2005; Behere and Bhise 2009), and the reality of agricultural work is often at odds with the societal perception of farming as a stress-free profession. Nevertheless, when asked to rate how satisfied they are with their occupation, farmers tend to give favorable responses, and farming is often highly ranked in large-scale job satisfaction surveys. Many farmers view their occupation not only as a job but a “lifestyle” (Schroeder et al. 1985; Vayro et al. 2020) and consider work satisfaction to be an important motivator alongside economic considerations (Coughenour and Swanson 1988; Willock et al. 1999). This feeling is seemingly even more pronounced among farmers involved in SFSCs (Feagan and Henderson 2009; Bruce 2019; Lioutas and Charatsari 2020). Thus, evaluating the relationship between the work environment of direct-market farmers and job satisfaction calls for an integrated framework that accounts for the farm sector’s unique characteristics.

### Defining short food supply chains

Presently, there is no commonly agreed-upon definition of SFSCs among scholars and practitioners (Paciarotti and Torregiani 2020). Nonetheless, the term is generally thought to encompass a range of marketing channels from direct-to-consumer outlets (farm kiosks, farmers’ markets, CSA initiatives, U-picks, internet sales, etc.) to intermediated food channels (sales to retailers, hotels, restaurants, and other places that market directly to consumers).

SFSCs are sometimes viewed as one component of the “alternative” food movement (Ilbery and Maye 2005; Qazi and Selfa 2005; Sonnino and Marsden 2006; Maxey 2007; Wilson 2013; Cleveland et al. 2014; Le Velly 2019), which operates “parallel to and mostly in opposition to” (Bui et al. 2019, p. 2) the mainstream industrial food system based on elongated supply chains. Conceptually, SFSCs constitute innovative forms of agricultural marketing that can facilitate the transition toward a more sustainable food system (Bui et al. 2019), one based on embedded community relationships (Hinrichs 2000; Murdoch et al. 2000) and values of trust (Venn et al. 2006). Put differently, these channels seek to “re-socialize” and “re-spatialize” the food landscape (Renting et al. 2003) by bringing producers and consumers closer together. In this sense, SFSCs are capable of “activating” geographical and social connections among community food stakeholders (Eriksen 2013; Kemkes and Akerman 2019).

### A multidimensional model of farmer satisfaction

According to Renting et al. (2003), the organization of SFSCs departs from conventional, market-based assumptions about food production and distribution. Direct-market farmers of course earn a livelihood from their work (Watts et al. 2005), meaning economic considerations cannot be overlooked. However, rather than guided by the market’s “invisible hand,” SFSCs are shaped through stakeholder relationships (Renting et al. 2003). Consequently, any analysis of occupational satisfaction among direct-market farmers must account for both economic and non-economic factors.

To develop our framework, we borrowed from Paugam’s (2000) analytical model, which posits that workers behave, simultaneously, as:

- *Homo faber* (referring to “the act of working itself and to the fulfillment it brings,” p. 44),
- *Homo sociologicus* (referring to the fact that “all work is carried out in a social context,” p. 44), and
- *Homo economicus* (referring to the pursuit of work for economic ends)*.*

For each typology, we discuss how Paugam’s framework can be used to analyze work satisfaction among farmers in SFSCs. The defining features of all three categories as they relate to direct farm marketing according to the literature are summarized in Table 1. Each feature has been worded to reflect a positive impact on occupational satisfaction.

**Table 1:** Categories and features of occupational satisfaction among direct-market farmers

Work enjoyment (*homo faber*)	Social satisfaction (*homo sociologicus)*	Economic satisfaction (*homo economicus)*
- The nature of the work itself (task enjoyment, skills development, low stress, few time-consuming tasks, low physical effort) (Dupré et al. 2017; Charatsari et al. 2019; Mundler and Jean-Gagnon 2020) - Freedom of initiative (control over production and farming methods, simplified management) (Galt et al. 2016; Paul 2019; Dupré et al. 2017)	- Interactions with clients (enjoyable relations with customers, meeting consumer expectations) (Hinrichs 2000; Sage 2003; Morris and Kirwan 2011; Mundler and Laughrea 2016) - Interactions with other farmers (recognition by and cooperation with other producers, collective action, manageable levels of competition) (Lawson et al. 2008; Chiffoleau 2009) - Image (perception of the farm in the community) (Marsden et al. 2000; Sonnino and Marsden 2006)	- Financial recognition (control over prices, satisfactory earnings) (Govindasamy et al. 1999; Hardesty and Leff 2010; Mundler and Jean-Gagnon 2020) - Financial health (economic performance) (Galt 2013; Lee et al. 2020) - Stability (secure market outlets) (Mundler and Laughrea 2016; Hatanaka 2020)

#### Enjoying work: the case of homo faber

Since SFSCs involve few or no middlemen, participating farmers are often more autonomous and have a greater ability to set prices independently. At the same time, research suggests that asymmetric market relations among stakeholders, a defining feature of the industrial food system, can also exist in SFSCs. For instance, Hinrichs (2000) noted that consumers in direct sales channels have greater market power than producers. In the case of CSA initiatives, farmers tend to price their food boxes based on their estimation of consumers’ willingness to pay. However, the risk of CSA members not renewing their subscriptions severely restricts the ability of producers to independently set prices (Cooley and Lass 1998; Paul 2019). Additionally, some studies have noted a tendency among direct-market farmers to engage in “self-exploitation,” for example, by accepting lower earnings in response to competition (Galt et al. 2016). Likewise, the high workloads that farmers in SFSCs take on can undermine occupational satisfaction (Dupré et al. 2017; Mundler and Jean-Gagnon 2020).

On the other hand, farmers who pursue direct marketing often report feeling a sense of pride in being able to use their skills to feed people and local communities. Conventional farmers undeniably also take pride in their work, although usually for different reasons. For instance, according to a study conducted in the Canadian province of Saskatchewan, export-oriented producers indicated that “feeding the world” was a source of pride for them (Beingessner and Fletcher 2020).

From a competency standpoint, farmers entering SFSCs need to possess certain skills to be successful, such as the ability to communicate effectively with clients. When such skills are lacking, it can lead to stress, lower productivity, and less interest in direct marketing (Charatsari et al. 2019). At the same time, studies show that participation in SFSCs can itself help farmers develop these critical abilities (Martinez et al. 2010; Kneafsey et al. 2013; Eugenio et al. 2017; Carbone 2018; Pereira et al. 2018; Sellitto et al. 2018; de Mansoldo et al. 2019). Direct-market farmers also tend to have higher levels of educational attainment compared to conventional producers, meaning many of them enter SFSCs already having developed a useful skillset (Uematsu and Mishra 2011; Bruce 2019).

#### Better together: the case of homo sociologicus

It has been shown that alternative food systems generally and SFSCs specifically enable farmers to build their confidence, develop connections with other stakeholders, and create community-level social capital (Kneafsey et al. 2013). For instance, in a study of CSA programs, Dufour et al. (2011) noted high levels of occupational satisfaction among participating producers, which was attributed to the quality of the relationships developed (both among producers and between producers and consumers), the enjoyment derived from collaborating on a joint project, and community social recognition. However, the study focused on a specific direct-marketing scheme, namely CSA food boxes, and, thus, it is unclear to what extent the findings apply to SFSCs more broadly. While cooperation is a defining feature of SFSCs, recent research suggests direct-market farmers also compete among themselves for clients (Connolly and Klaiber 2019). Furthermore, studies from the United States indicate that sales from direct-to-consumer outlets (such as CSA initiatives) have slowed in recent years, although sales from intermediated channels continue to grow (Printezis and Grebitus 2018; Dimitri and Gardner 2019; Boys and Fraser 2019; Feenstra et al. 2019).

#### Doing business: the case of homo economicus

In Paugam’s framework, *homo economicus* embodies one of the three facets of job satisfaction. While Paugam showed that satisfaction hinges on more than just pay, economic factors are still considered, especially in public debates, as one of the most – if not the most – important determinants of occupational satisfaction.

According to Paugam (2007), work satisfaction in this category is measured by the degree to which a worker is satisfied with his or her salary and promotion prospects. In the context of SFSCs, this definition, with its focus on employee compensation, is inadequate since direct-market farmers are self-employed and any salary is self-paid or decided at the household level. Dufour et al. (2011), however, adapted Paugam’s classification scheme to reflect the economic environment in which direct-market farmers operate. According to their model, compensation levels, the regularity of income, farm performance, and risk factors can be used to measure economic satisfaction among producers in SFSCs.

## Materials and methods

Data were collected during the winter of 2019 using a web survey administered across all ten Canadian provinces. Some provinces were later grouped into geographical regions (this was the case for the Prairie provinces and Atlantic provinces) to account for low response rates in certain areas of the country. Since there are no actual lists of direct-market farmers, participants were selected using nonprobability sampling. For practical purposes, we consider SFSCs to be marketing channels that involve no more than one middleman, which is in line with the definition adopted by many researchers and policymakers in Canada and Europe (European Commission 2013; Malak-Rawlikowska et al. 2019). Producers who sold through such outlets at the time of the survey were considered to meet the inclusion criteria. After initially administering the survey, we sent contacted farmers a follow-up invitation to participate if they had not already done so. In total, we obtained a 16.7% response rate, consisting of 904 returned questionnaires. Of these, 613 were complete or nearly complete and were used for the present analysis. We used pairwise deletion to account for any missing data.

The survey was administered online through the LimeSurvey platform and included socio-economic questions, as well as questions about farm characteristics and the work environment. Most studies view participation in SFSCs through a binary lens (a producer either pursues direct marketing or does not) rather than measuring a farmer’s *degree* of involvement. However, in this study, sales from direct marketing as a percentage of total farm sales (hereafter referred to as SFSCshare) were used to measure participation levels.

We measured farmer satisfaction in two ways. First, for each of the three categories of satisfaction considered (work enjoyment, social satisfaction, economic satisfaction) respondents were asked to indicate the extent to which they agreed or disagreed with a series of statements using a five-point Likert scale (from strongly disagree = 1 to strongly agree = 5). Second, respondents were presented with a single statement intended to measure their overall level of satisfaction in each category, this time using a ten-point Likert scale in which only the endpoints were labeled (strongly disagree = 1, strongly agree = 10). Specifically, the survey asked farmers to rate their level of agreement or disagreement with the following three statements: “overall, I am fully satisfied with my work” (work enjoyment); “overall, I feel that my work is well recognized” (social satisfaction); and “overall, I am fully satisfied with my economic situation” (economic satisfaction). Surveyed farmers were also queried about their values and their perception of the role of agriculture. The pre-test questionnaire was administered to ten farmers in different provinces who provided feedback, enabling us to improve the wording of certain questions.

We used Cronbach’s alpha and item-total correlation to test for scale reliability, with the results indicating that scores for work enjoyment (7 items, Cronbach’s alpha = 0.83), social satisfaction (6 items, Cronbach’s alpha = 0.74), and economic satisfaction (5 items, Cronbach’s alpha = 0.71) were statistically correlated within each category. Further analysis using item-response theory (DeVellis 2017) did not reveal any major issues with item quality.

Two models were developed to test for correlations between SFSCshare and farmer satisfaction in each of the three domains considered, and calculations were carried out using the statistical software package Stata. To isolate the effect of SFSCshare, we included in both models a series of socio-economic and geographic control variables. We first tested an ordinary least squares (OLS) regression model by treating the five-point Likert scale as an interval variable (Carifio and Perla 2008; Harpe 2015). Here, the dependent variable was defined as the mean response to the five-point items associated with each category. To determine the robustness of the results obtained, we then ran a logistic regression in which the ten-point Likert item measuring overall satisfaction was transformed into a binary variable. This was done by assigning a value of 0 (indicating a general lack of satisfaction) to scores between 1 (“strongly disagree”) and 5 and a value of 1 (indicating general satisfaction) to scores over 5 (with a value of 10 signifying that the respondent “strongly agreed” with the statement). We were unable to subsequently test an ordinal logistic model using overall satisfaction scores because the assumption of a proportional odds ratio was violated. Nevertheless, our decision to transform the ordinal scores into a binary variable is consistent with previous work on job satisfaction (D’Addio et al. 2007).

Mathematically, our model for each job satisfaction category is as follows:

$${Y}_{i}=A+\sum {B}_{j}{X}_{j}+u$$

where:

${Y}_{i}$: An interval (OLS model) or binary (logistic model) measurement of work enjoyment, social satisfaction, and economic satisfaction.

${X}_{j}$: A vector of regressors (including SFSCshare).

${B}_{j}$_:_ A vector of coefficients.

$u$: The error term of the regression model.

$A$: The intercept of the regression model.

Since farmers decide how much to participate in direct-marketing channels (which implies a form of self-selection), we conducted a Durbin-Wu-Hausman test for endogeneity on the variable SFSCshare (Nakamura and Masao 1998). A correlation between SFSCshare and the error term would violate one of the key assumptions of the OLS method and lead to biased or inconsistent estimates (Wooldridge 2006; Bascle 2008; Greene 2009; Antonakis et al. 2010). The instrumental variable for the test was taken from one section of the survey where respondents were asked to indicate the extent to which they agreed or disagreed (using a five-point scale) with the notion that the role of agriculture is to earn foreign currency through exports. We chose this variable based on the assumption it would be (negatively) correlated with participation in SFSCs but not with farmer satisfaction. The null hypothesis being tested in each case is that the variable SFSCshare is exogenous. The results (Table 2) for each dimension of occupational satisfaction found no evidence of endogeneity (at the five percent significance level), thus confirming our model’s validity.

**Table 2:** Durbin-Wu-Hausman endogeneity test for the main explanatory variable (SFSCshare)

Work enjoyment	Social satisfaction	Economic satisfaction
Durbin (score) chi2(1) = 0.863 (*p* = 0.353)	Durbin (score) chi2(1) = 0.863 (*p* = 0.353)	Durbin (score) chi2(1) = 1.733 (*p* = 0.188)
Wu-Hausman F(1.572) = 0.809 (*p* = 0.369)	Wu-Hausman F(1.572) = 0.809 (*p* = 0.369)	Wu-Hausman F(1.572) = 1.627 (*p* = 0.203)

The next section presents our results. We examine the descriptive statistics obtained from the survey, followed by the results of the two regression models.

## Findings

### Descriptive statistics

The independent variables used in our model are presented in Table 3 (interval variables) and Table 4 (categorical variables). In addition to our main variable of interest (SFSCshare), we hypothesize that occupational satisfaction among direct-market farmers is influenced by other factors, such as the work environment and a producer’s socio-economic profile.

**Table 3:** Characteristics of surveyed direct-market farmers (interval variables) (*N* = 613)

Variable name	Description	Mean	Standard deviation
SFSCshare	Sales from direct marketing as a percentage of total farm sales	80.06	29.40
INTERMEDshare	Sales from intermediated channels as a percentage of total direct marketing sales	19.42	26.19
CROPshare	Sales from crops as a percentage of total farm sales	57.47	43.66
NUMBERCHANNELS	Number of direct-market channels used	3.44	1.46
AREA	Total cultivated area (ha)	52.78	183.33
OPERATORS	Number of farm operators	2.06	1.064
AGE	Respondent’s age	48.38	12.95
WORKHOURS	Number of annual hours worked by the main farm operator	2000.01	1041.74

**Table 4:** Characteristics of direct-market farmers (categorical variables) (*N* = 613)

Variable name	Description	Category	Frequency	Percentage
GREVENUE	Gross revenue	Less than $10,000^a^	49	7.99
		$10,000 to $50,000	170	27.73
		$50,000 to $100,000	110	17.94
		$100,000 to $250,000	133	21.70
		$250,000 to $500,000	75	12.23
		$500,000 to $1,000,000	39	6.36
		More than $1,000,000	37	6.04
NREVENUE	Net revenue	Negative return	116	18.92
		$0 to $20,000	229	37.36
		$20,000 to $40,000	145	23.65
		$40,000 to $75,000	67	10.93
		$75,000 to $150,000	40	6.53
		More than $150,000	16	2.61
GENDER	Gender (Female, Male)	Female	302	49.27
HIGHEREDUC	Attended college or university	Yes	521	84.99
TRAINING	Trained in agriculture	Yes	218	35.56
FARMORIGIN	Origin of the farm	Inherited farm	111	18.11
		Start-up	461	75.20
		Third-party transfer	41	6.69
PREVIOUSOCCUPATION	Prior occupation before farming	Yes	477	77.81
REGION	Geographic region	Atlantic provinces^b^	64	10.44
		Quebec	231	37.68
		Ontario	114	18.60
		Prairie provinces^c^	79	12.89
		British Columbia	125	20.39
FARMSETTING	Farm location	Suburban	94	15.33
		Rural	496	80.91
		Urban	23	3.75
EMPLOYEES	Hired farmworkers	Yes	384	62.64
VOLUNTEERS	Volunteers	Yes	368	60.03
OTHERINCOME	Other sources of income	Yes	459	74.88
PROCESSING	On-farm processing	Yes	256	41.76
AGRITOURISM	Receives visitors to the farm	Yes	316	51.55
ORGANIC	Certified organic or in transition	Yes	215	35.07

^a^Figures are expressed in Canadian dollars

^b^New Brunswick, Nova Scotia, Prince Edward Island, and Newfoundland and Labrador

^c^Alberta, Saskatchewan, and Manitoba

Overall, survey respondents were highly involved in SFSCs, generating an average of 80% of total farm revenues from direct marketing. On average, just under 20% of direct sales were derived from intermediated food channels. Surveyed producers typically operated small- or medium-sized farms, grew vegetables, and used an average of three direct-market channels. One or two operators typically managed each farm, with the main operator assuming most of the work.

Table 4 highlights the diverse pathways into farming that respondents pursued. Most participants had started their farm (rather than taking ownership of an already existing one), were highly educated (85% had attended college or university), and had practiced another occupation before becoming a farmer. The use of hired labor and volunteers was common among respondents. Most of them also had other sources of income besides farming, challenging the notion that work performed on farms in SFSCs carries few opportunity costs (Corsi et al. 2018). In terms of net revenue, while most surveyed producers could be classified as low or medium earners, nearly one-fifth reported negative earnings, which is problematic given that direct-market farmers typically use their net revenues to pay themselves a salary.

About half of respondents provided guest accommodations and/or offered visitors the opportunity to engage in experiential activities. Compared to the national average, a disproportionate number of surveyed farmers also practiced organic farming. Furthermore, while most farmers marketing through conventional supply chains are men, almost half of respondents were women, which echoes the results of previous studies that point to a large presence of female producers in SFSCs, agritourism, and organic farming (DeLind and Ferguson 1999; McGehee et al. 2007; Tijani and Yano 2007; Trauger 2004; Trauger et al. 2010; Finan 2011; Jarosz 2011; Sumner and Llewelyn 2011; Annes and Wright 2015).

**Fig. 1:**
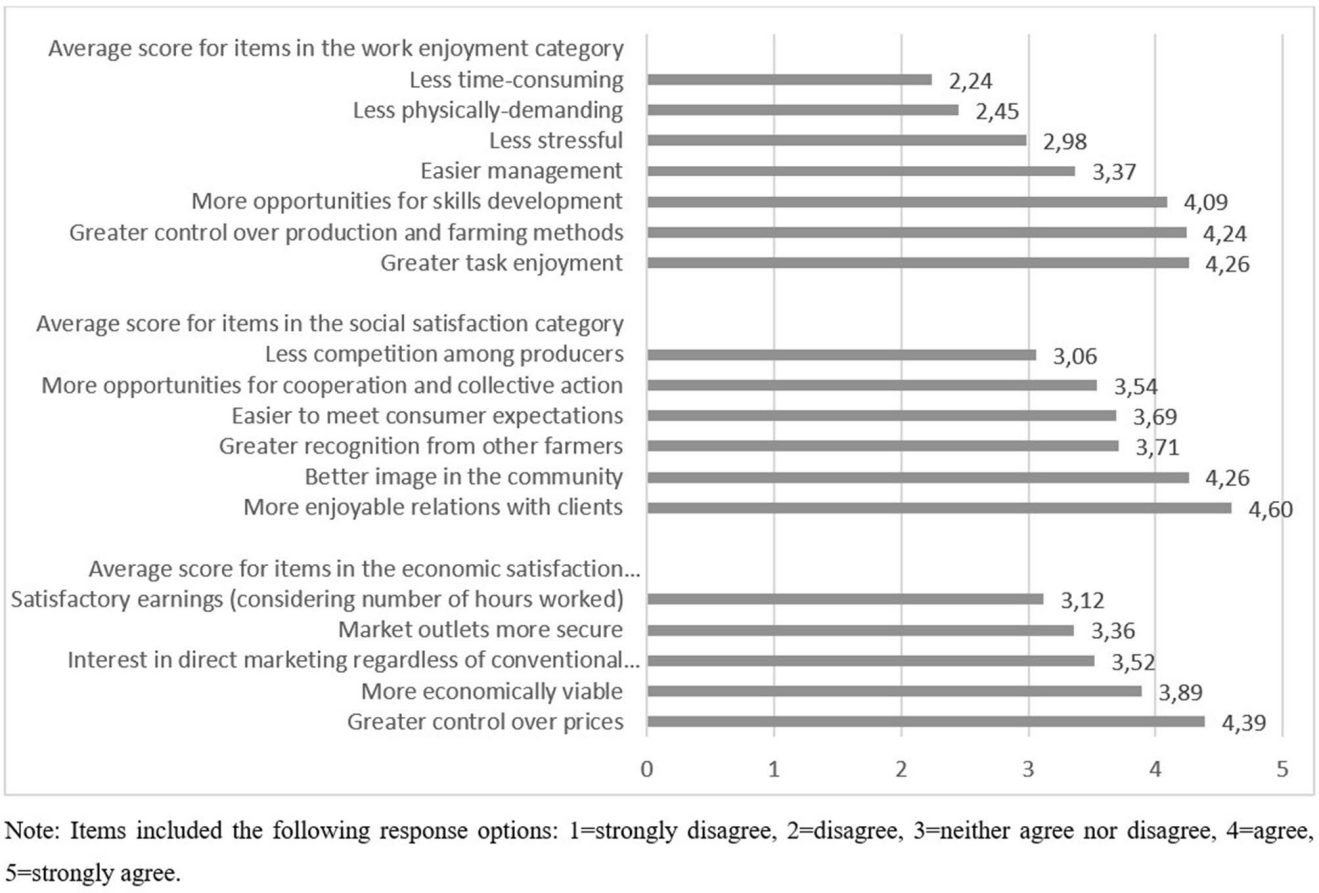
Mean item score by domain of occupational satisfaction

Figure 1 presents the mean value for each item (measured on a five-point Likert scale) grouped by category of occupational satisfaction. On average, respondents gave low scores to statements suggesting that participating in SFSCs is less stressful, less physically challenging, and less time consuming, indicating that direct-market farmers often take on considerable workloads. On the other hand, items connecting SFSC participation with greater levels of empowerment, autonomy, and task enjoyment generally received higher scores, as did items measuring social satisfaction, which confirms previous findings on the benefits of direct marketing. Likewise, items in the economic satisfaction category on average received favorable scores. The ability of farmers to set prices independently was the most strongly acknowledged economic advantage of SFSCs, followed by the belief that direct marketing enables producers to develop economically viable projects. However, respondents were generally not satisfied with the earnings generated through SFSCs considering the time commitment that such channels require, which suggests many of the economic benefits associated with direct marketing come with a trade-off.

For each category, Table 5 presents the mean of the five-point items, as well as the mean for overall satisfaction (i.e., the single ten-point item). Regardless of the measurement instrument used, average satisfaction scores were positive across all categories (above three for the five-point items and above five for the ten-point items). However, the category rankings changed depending on the measurement tool. For instance, work enjoyment obtained the highest score when comparing the mean of the five-point items but received the lowest overall satisfaction score (as measured by the single item). The shift could be due to the equal weight attributed to each five-point item when calculating the mean, whereas the single item implicitly allows respondents to decide how much importance to attribute to each factor when making an overall determination of their satisfaction. Identifying the most appropriate tools for measuring job satisfaction remains a topic of ongoing debate among researchers.

**Table 5:** Average satisfaction scores among direct-market farmers

Job satisfaction category	Measurement	*N*	Likert scale	Mean	Standard deviation
Work enjoyment	7 items	613	1–5	3.38	0.76
	Single item^a^	612	1–10	8.11	1.52
Social satisfaction	6 items	613	1–5	3.81	0.60
	Single item^a^	612	1–10	7.84	1.83
Economic satisfaction	5 items	613	1–5	3.66	0.69
	Single item^a^	610	1–10	6.10	2.31

^a^For the single items, only the endpoints were labeled: 1 = strongly disagree, 10 = strongly agree

### Models estimates

Table 6 shows the OLS regression results for each domain of job satisfaction. As noted previously, the dependent variable was defined as the mean response to the five-point items for each category (see Tables 1 and 5 and Fig. 1 for details regarding scale construction). Each regression identifies the factors contributing to occupational satisfaction in the domain considered. As we mentioned before, the main variable of interest is SFSCshare, namely the percentage of total farm sales attributable to direct marketing.

**Table 6:** OLS regression for occupational satisfaction among direct-market farmers

	Work enjoyment (*N* = 611)		Social satisfaction (*N* = 611)		Economic satisfaction (*N* = 611)	
	Coefficient	Standard error^a^	Coefficient	Standard error^a^	Coefficient	Standard error^a^
SFSCshare	0.004***	(0.001)	0.003**	(0.001)	0.004***	(0.001)
INTERMEDshare	−0.005***	(0.001)	−0.003**	(0.001)	−0.005***	(0.001)
CROPshare	0.002*	(0.001)	0.00008	(0.00058)	0.001	(0.001)
NUMBERCHANNELS	−0.043	(0.023)	0.001	(0.018)	−0.027	(0.019)
AREA	−0.00009	(0.00012)	−0.00017	(0.00009)	−0.00008	(0.00014)
OPERATORS	0.058	(0.032)	0.054*	(0.026)	0.021	(0.030)
AGE	0.006*	(0.002)	0.001	(0.002)	0.007**	(0.002)
WORKHOURS	−0.00004	(0.00003)	−0.00002	(0.00003)	−0.00003	(0.00003)
GREVENUE = $10,000 to $50,000	−0.162	(0.118)	0.050	(0.094)	−0.064	(0.120)
GREVENUE = $50,000 to $100,000	−0.364**	(0.134)	0.037	(0.104)	0.019	(0.131)
GREVENUE = $100,000 to $250,000	−0.443**	(0.146)	−0.054	(0.113)	−0.169	(0.143)
GREVENUE = $250,000 to $500,000	−0.466**	(0.166)	−0.157	(0.135)	−0.137	(0.158)
GREVENUE = $500,000 to $1,000,000	−0.647***	(0.197)	−0.104	(0.176)	−0.231	(0.190)
GREVENUE = More than $1,000,000	−0.244	(0.211)	0.212	(0.164)	0.213	(0.206)
NREVENUE = $0 to $20,000	0.193*	(0.082)	0.243***	(0.071)	0.394***	(0.080)
NREVENUE$20,000 to $40,000	0.234*	(0.104)	0.274***	(0.082)	0.489***	(0.094)
NREVENUE = $40,000 to $75,000	0.183	(0.127)	0.158	(0.105)	0.576***	(0.116)
NREVENUE = $75,000 to $150,000	0.146	(0.152)	0.129	(0.124)	0.531***	(0.141)
NREVENUE = More than $150,000	−0.253	(0.218)	−0.295	(0.166)	0.069	(0.238)
GENDER = Female	0.099	(0.059)	0.168***	(0.048)	0.108*	(0.055)
HIGHEREDUC = Yes	−0.126	(0.084)	0.012	(0.069)	−0.142	(0.079)
TRAINING = Yes	−0.142*	(0.066)	−0.132*	(0.054)	−0.088	(0.057)
FARMORIGIN = Start-up	−0.014	(0.079)	0.018	(0.067)	−0.020	(0.074)
FARMORIGIN = Third-party transfer	0.107	(0.135)	0.025	(0.105)	0.113	(0.129)
PREVIOUSOCCUPATION = Yes	0.064	(0.074)	0.022	(0.059)	−0.019	(0.064)
REGION = Quebec	−0.085	(0.092)	−0.115	(0.082)	−0.040	(0.092)
REGION = Ontario	0.043	(0.105)	−0.083	(0.087)	−0.101	(0.104)
REGION = Prairie provinces	−0.030	(0.115)	−0.030	(0.093)	−0.089	(0.107)
REGION = British Columbia	−0.129	(0.105)	−0.025	(−0.025)	−0.158	(−0.158)
FARMSETTING = Rural	0.037	(0.077)	0.026	(0.058)	0.051	(0.069)
FARMSETTING = Urban	0.144	(0.187)	−0.072	(0.174)	0.113	(0.181)
EMPLOYEES = Yes	−0.003	(0.072)	0.132*	(0.059)	−0.021	(0.066)
VOLUNTEERS = Yes	0.112	(0.059)	0.125*	(0.049)	0.129*	(0.058)
OTHERINCOME = Yes	−0.027	(0.073)	0.022	(0.061)	−0.089	(0.068)
PROCESSING = Yes	0.049	(0.062)	0.061	(0.053)	0.027	(0.055)
AGRITOURISM = Yes	0.059	(0.058)	0.089	(0.049)	0.085	(0.054)
ORGANIC = Yes	−0.016	(0.063)	0.053	(0.053)	0.075	(0.060)
_cons	3.009***	(0.294)	3.078***	(0.241)	2.937***	(0.274)

*p < 0.05, **p < 0.01, ***p < 0.001

^a^Robust standard errors are reported

The results indicate that SFSCshare is positively correlated with levels of occupational satisfaction across all three domains. In other words, the more farmers pursue direct marketing the more satisfied they are with their work and the higher their rates of economic and social satisfaction. Somewhat unexpectedly, a greater use of intermediated food channels had the opposite effect on job satisfaction in each area. These seemingly contradictory findings suggest that many of the occupational benefits associated with SFSCs can only be obtained through direct-to-consumer channels.

Net annual revenue (a measure of household farm income) was correlated with higher levels of job satisfaction in every category when comparing farms in the $0–$40,000 brackets with the reference group (farms earning a negative return). However, above this threshold, the effect dissipated in certain domains. Specifically, net earnings between $40,000 and $150,000 did not lead to greater work enjoyment or social satisfaction, although the positive effect on economic satisfaction remained significant. These results suggest that using family labor on farms in SFSCs involves an opportunity cost, which seemingly contradicts the claims made by Corsi et al. (2018). If net revenue is defined as the farm earnings of non-salaried family members (including the main farmer), then, in the absence of opportunity costs, an increase in net revenue would likely not have a positive impact on economic satisfaction. Indeed, if there were no opportunity costs for farm work, then the effect of higher net revenues on economic satisfaction would probably be neutral, not positive. Since our results indicate a positive correlation, it is reasonable to assume that farmers in SFSCs allocate their time between farming and off-farm work based on the respective returns from each activity.

Interestingly, gross income was negatively correlated with work enjoyment (here the reference category was farms with gross incomes between $0 and $10,000). The negative effect was significant across all revenue classes between $50,000 and $1 million, indicating that direct-market farmers in higher income brackets are more likely to undertake stressful, physically demanding, or time consuming activities that have a detrimental effect on work enjoyment. At the same time, gross income levels had no significant effect on social or economic satisfaction.

Another counterintuitive result was the negative impact of agricultural training, with trained farmers reporting lower average scores for work enjoyment and social satisfaction. However, the use of hired labor and volunteers and the number of farm operators were all positively correlated with social satisfaction. Many respondents reported that farm operators, volunteers, and employees all participate in production and post-production activities, such as distribution and marketing. This collective effort seemingly prevents farmers from feeling overwhelmed by the numerous interactions with clients and other stakeholders, since others can be relied upon to help, leading to a more positive social experience. Volunteer support also had a positive impact on economic satisfaction undoubtedly because the use of unpaid labor enables farmers in SFSCs to overcome certain financial constraints associated with direct marketing (Biewener 2016).

As for the other independent variables, a farmer’s age had a positive effect on work enjoyment and economic satisfaction, and being a female farmer was positively correlated with both social and economic satisfaction. In addition, crop sales expressed as a percentage of total farm sales was a positive predictor of work enjoyment (possibly because crop production involves simplified management).

Table 7 presents our second model, the logistic regression, in which we assess the robustness of the results obtained from the OLS model. As mentioned previously, the dependent variable for the logistic regression was created by transforming the single ten-point Likert item for each category (measuring overall satisfaction, see Table 5) into a binary variable. We expect that factors found to be statistically significant in the OLS model will remain so.

**Table 7:** Logistic regression for occupational satisfaction among direct-market farmers

	Work enjoyment (*N* = 555^a^)		Social satisfaction (*N* = 555^a^)		Economic satisfaction (*N* = 610)	
	Coefficient	Standard error	Coefficient	Standard error	Coefficient	Standard error
SFSCshare	0.017*	(0.008)	0.005	(0.005)	0.009*	(0.004)
INTERMEDshare	−0.015	(0.008)	−0.008	(0.005)	−0.011**	(0.004)
CROPshare	0.013	(0.007)	0.005	(0.004)	0.006*	(0.003)
NUMBERCHANNELS	−0.106	(0.154)	0.167	(0.110)	−0.032	(0.071)
AREA	−0.002	(0.001)	−0.00012	(0.00076)	0.00008	(0.00055)
OPERATORS	−0.093	(0.289)	0.154	(0.177)	0.212	(0.119)
AGE	−0.012	(0.020)	−0.003	(0.012)	0.024**	(0.008)
WORKHOURS	0.00013	(0.00024)	−0.00019	(0.00015)	−0.00013	(0.00010)
GREVENUE = $10,000 to $50,000	0.827	(0.744)	−0.818	(0.578)	0.225	(0.379)
GREVENUE = $50,000 to $100,000	0.888	(0.886)	0.160	(0.700)	0.839	(0.440)
GREVENUE = $100,000 to $250,000	0.570	(0.922)	−0.530	(0.708)	0.263	(0.479)
GREVENUE = $250,000 to $500,000	1.320	(1.402)	−0.094	(0.858)	0.122	(0.553)
GREVENUE = $500,000 to $1,000,000	−0.718	(1.274)	−0.596	(0.961)	0.359	(0.679)
GREVENUE = More than $1,000,000	−0.236	(1.510)	−0.041	(1.183)	0.551	(0.785)
NREVENUE = $0 to $20,000	3.095***	(0.674)	1.115**	(0.355)	1.205***	(0.259)
NREVENUE = $20,000 to $40,000	2.779**	(0.904)	1.117*	(0.470)	1.597***	(0.321)
NREVENUE = $40,000 to $75,000	2.702**	(0.994)	1.287*	(0.617)	2.477***	(0.445)
NREVENUE = $75,000 to $150,000	3.633*	(1.537)	1.854*	(0.883)	3.424***	(0.637)
NREVENUE = More than $150,000	–	–	–	–	3.593**	(1.186)
GENDER = Female	0.004	(0.503)	0.656*	(0.318)	0.099	(0.203)
HIGHEREDUC = Yes	−0.177	(0.624)	0.369	(0.370)	−0.225	(0.286)
TRAINING = Yes	0.076	(0.535)	−0.319	(0.334)	−0.083	(0.219)
FARMORIGIN = Start-up	−2.114*	(1.002)	−0.089	(0.462)	0.129	(0.309)
FARMORIGIN = Third-party transfer	–	–	–	–	0.201	(0.487)
PREVIOUSOCCUPATION = Yes	1.640**	(0.610)	0.656	(0.380)	−0.061	(0.265)
REGION = Quebec	1.659*	(0.828)	0.096	(0.599)	0.428	(0.348)
REGION = Ontario	0.218	(0.770)	−0.576	(0.615)	−0.100	(0.375)
REGION = Prairie provinces	2.323	(1.204)	−1.027	(0.635)	0.087	(0.408)
REGION = British Columbia	0.035	(0.035)	−0.768	(−0.768)	0.194	(0.194)
FARMSETTING = Rural	−0.441	(0.774)	0.048	(0.443)	0.363	(0.280)
FARMSETTING = Urban	−2.086	(1.247)	0.228	(0.916)	0.530	(0.598)
EMPLOYEES = Yes	0.128	(0.559)	0.078	(0.353)	0.135	(0.240)
VOLUNTEERS = Yes	0.259	(0.515)	−0.077	(0.306)	0.186	(0.206)
OTHERINCOME = Yes	−0.837	(0.759)	−0.173	(0.422)	−0.026	(0.257)
PROCESSING = Yes	0.010	(0.515)	−0.326	(0.321)	−0.200	(0.218)
AGRITOURISM = Yes	−0.162	(0.489)	0.226	(0.310)	0.226	(0.204)
ORGANIC = Yes	−0.754	(0.569)	0.346	(0.356)	−0.413	(0.224)
_cons	1.357	(2.418)	0.275	(1.464)	−3.578***	(0.999)

*p < 0.05, **p < 0.01, ***p < 0.001

^a^55 observations were dropped from the work enjoyment and social satisfaction models because earning more than $150,000 in net revenue and obtaining the farm through a third-party transfer was perfectly correlated with the dependent variable

Factors that prove significant in both models can be considered robust enough to explain job satisfaction among farmers in SFSCs. There were no changes in signs (except for an intercept), although certain factors found to be significant in the OLS regression were no longer so in the logistic regression. For instance, in the logistic model, being a female farmer and age only had a positive effect on social satisfaction and economic satisfaction, respectively. At the same time, certain variables whose coefficients were not statistically significant in the OLS regression became significant. For example, practicing a profession before farming and operating in the province of Quebec were both associated with higher levels of work enjoyment, while starting a new farm had the opposite effect.

Our main explanatory variable (SFSCshare) remained significant in the logistic model, positively correlating with work enjoyment and economic satisfaction, although the effect on social satisfaction disappeared. This does not imply though that farmers in SFSCs are dissatisfied with the social aspects of direct marketing. Indeed, as we saw in Fig. 1, the mean scores for items measuring social satisfaction were relatively high. Rather, the absence of an association suggests that social satisfaction among direct-market farmers does not significantly increase (or decrease) with more active involvement. We note, however, that greater participation in intermediated channels remained negatively correlated with economic satisfaction in the logistic model, again underscoring that direct-to-consumer outlets may hold certain unique advantages over other SFSCs. Finally, the net revenue classes broadly captured the same effects as the first model, with higher net earnings continuing to be positively associated with work enjoyment and social and economic satisfaction (compared to farms with negative returns).

## Discussion and conclusion

Our study applied a job satisfaction framework to organize the potential benefits of direct farm marketing into three categories of occupational satisfaction: work enjoyment, social satisfaction, and economic satisfaction. We then proceeded to test the extent to which direct-market farmers were satisfied with each category and whether reported levels of satisfaction were influenced by the degree of participation in SFSCs.

Regardless of the instrument used (multi- or single-item questions), the average scores in each domain were high, echoing previous findings on the social and economic advantages of direct marketing (Kneafsey et al. 2013). However, a closer examination revealed that certain aspects of the work environment on farms involved in SFSCs negatively affected satisfaction scores. For instance, the often stressful, physically demanding, and time consuming nature of direct marketing lowered the average score for work enjoyment, confirming what prior studies have found, namely that excessive workloads and labor constraints are critical problems for direct-market farmers (Galt 2013; Dupré et al. 2017). Future research should be conducted to explore how farmers in SFSCs deal with such challenges.

As Fig. 1 showed, the item associating SFSCs with greater cooperation received the second-lowest average score in the social satisfaction category. While social embeddedness is often viewed as an inherent feature of SFSCs (Hinrichs 2000; Winter 2003; Sage 2003; Sonnino 2007; Morris and Kirwan 2011), our survey results suggest stakeholder collaboration is not necessarily greater in such channels compared to conventional supply chains. Prior research has certainly found that direct-market farmers are more likely to cooperate, and numerous joint initiatives within SFSCs have been successfully implemented (Chiffoleau 2009). However, it would appear from our findings that many farmers who practice direct marketing still face individual and systemic barriers that hinder the potential for greater collective action (Kessari et al. 2020).

Among the items tied to economic satisfaction, respondents were least satisfied with the income earned from direct sales. This outcome is unsurprising since earnings from SFSCs are usually modest compared to the number of hours farmers spend producing and marketing through such channels. Prior studies have also documented similar cases of financial dissatisfaction among CSA-involved farmers (Tegtmeier and Duffy 2005; Feagan and Henderson 2009; Paul 2019).

In both regression models, the share of total farm sales attributable to direct selling (SFSCshare) had a positive effect on work enjoyment and economic satisfaction. Social satisfaction was also positively correlated with this variable, although only in the OLS model. Other explanatory factors that proved robust in both models were net revenue levels (positive across all categories), gender (positive effect on social satisfaction), age (positively correlated with economic satisfaction), and the share of direct sales from intermediated channels (negative effect on economic satisfaction).

All in all, these findings challenge core beliefs about the socially embedded nature of SFSCs. While surveyed farmers, on average, gave favorable scores to items associating direct marketing with various social benefits, the degree of involvement in SFSCs (as measured by SFSCshare) had no statistical effect on social satisfaction. One possible explanation for this discrepancy could be that farmers enjoy interacting with other food stakeholders regardless of whether such exchanges occur in a direct marketing context. In this regard, our findings support previous studies that criticize the notion of a socially embedded/dis-embedded dichotomy between SFSCs and the conventional food system (Hedberg II and Zimmerer 2020).

As noted previously, net revenue and gender (being a female farmer) both had a positive effect on social satisfaction, which suggests the social benefits of SFSCs are tied to farm performance and gendered differences in skills and professional orientation (Hinrichs 2000; Jarosz 2011). Indeed, as Zirham and Palomba (2016, p. 377) noted in their study of female producers in SFSCs: “the female contribution is particularly important since […] women are more able to integrate the community and build social ties, sense of trust and reciprocity: woman, that is, bring back the production and consumption operations to a social human dimension, in which manufacturers and their activities are no longer isolated entities, but are visible to society.”

The present study adds to the growing literature on SFSCs in three important ways. Firstly, from a theoretical perspective, we utilized a job satisfaction framework to determine whether the benefits attributed to SFSCs are real, drawing upon research methods and perspectives from psychology, sociology, and economics. Rather than adopting a systemic approach (one that incorporates all relevant SFSC stakeholders), our research focused on the impact that direct marketing has on producers specifically. Future studies could employ a similarly focused approach to examine the potential benefits that consumers derive from involvement in SFSCs.

Secondly, our study proposes a new way of measuring the impact of alternative food systems at the farm level using quantitative or mixed methods. The results obtained also confirm that scale measurements are promising tools for analyzing the relational, economic, and psychological features of socially embedded food networks.

Thirdly, our findings, which indicate generally high levels of occupational satisfaction among direct-market farmers, contribute to a better understanding of the benefits of SFSCs. Future research, however, should further examine the relation between farmer participation in SFSCs and social satisfaction since the results we obtained were not significant across both models. From a gender perspective, being a woman farmer was correlated with greater social satisfaction, which echoes the results of previous scholarship on active female participation in direct farm marketing. Nevertheless, the exact mechanisms by which gender influences occupational satisfaction and the values embedded within SFSCs (DeLind and Ferguson 1999) remains an open question. Likewise, the significant effect of net revenue on farmer satisfaction observed in our study highlights the need for more research on the use and organization of family labor on farms engaged in SFSCs.

In terms of limitations, our findings, while interesting, do not provide decisive conclusions about what contributes to work satisfaction (or dissatisfaction) among direct-market farmers. While our results are consistent with the findings of previous studies, it is important to bear in mind that the data was collected through non-probabilistic sampling. Ideally, further research should be conducted based on a randomly selected sample. Tests could also be carried out to isolate and compare the impact of different direct sales channels (farmers’ markets, CSA initiatives, etc.) on job satisfaction.

Nevertheless, our study has the merit of quantitively testing various observations from prior qualitative research on farmer satisfaction within SFSCs. It also underscores the benefits of not focusing on a limited geographical area or a single sales channel when examining the impact of direct marketing. At the same time, the correlations identified in our models do not imply causation and should be further tested by examining a wider range of social contexts or by using analytical frameworks for studying work satisfaction derived from other social sciences. Likewise, future research could enrich our findings by formulating more specific questions for each domain of satisfaction or direct marketing channel. In this sense, our study opens the door to a new and promising area of research with important political, economic, and social implications for farms involved in SFSCs.

## Abbreviations

SFSCs: Short Food Supply Chains
CSA: Community-Supported Agriculture
OLS: Ordinary Least Squares

## Footnotes

1 In this article, we use the terms short food supply chains and direct farm marketing interchangeably.
